# The association between brain-derived neurotrophic factor gene polymorphism and migraine: a meta-analysis

**DOI:** 10.1186/s10194-017-0725-2

**Published:** 2017-02-02

**Authors:** Xiaoying Cai, Xiaolei Shi, Ximeng Zhang, Aiwu Zhang, Minying Zheng, Yannan Fang

**Affiliations:** 1 Guangdong Key Laboratory for Diagnosis and Treatment of Major Neurological Diseases, Department of Neurology, National Key Clinical Department and Key Discipline of Neurology, The First Affiliated Hospital, Sun Yat-sen University, Guangdong, 510080 China; 2 Department of Neurology, The First Affiliated Hospital, Yijishan Hospital of Wannan Medical College, Anhui, 241001 China; 3 Department of Neurology, The First Affiliated Hospital, Soochow University, No. 188 Shizi Road, Jiangsu, 215000 China

**Keywords:** BDNF, rs6265, rs2049046, Val66Met polymorphism, Migraine

## Abstract

**Background:**

Migraine is a recurrent headache disease related to genetic variants. The brain-derived neurotrophic factor (BDNF) gene rs6265 (Val66Met) and rs2049046 polymorphism has been found to be associated with migraine. However, their roles in this disorder are not well established. Then we conduct this meta-analysis to address this issue.

**Methods:**

PubMed, Web of Science and Cochrane databases were systematically searched to identify all relevant studies. Odds ratio (OR) with corresponding 95% confidence interval (CI) was used to estimate the strength of association between BDNF gene rs6265 and rs2049046 polymorphism and migraine.

**Results:**

Four studies with 1598 cases and 1585 controls, fulfilling the inclusion criteria were included in our meta-analysis. Overall data showed significant association between rs6265 polymorphism and migraine in allele model (OR = 0.86, 95%CI: 0.76–0.99, *p* = 0.03), recessive model (OR = 0.84, 95%CI: 0.72–0.98, *p* = 0.03) and additive model (GG vs GA: OR = 0.85, 95%CI: 0.72–1.00, *p* = 0.04), respectively. We also found significant association between rs2049046(A/T) polymorphism and migraine in allele model (OR = 0.88, 95%CI: 0.79–0.98, *p* = 0.02), recessive model (OR = 0.80, 95%CI: 0.67–0.96, *p* = 0.02) and additive model (AA vs TT: OR = 0.72, 95%CI: 0.57–0.92, *p* = 0.008; AA vs AT: OR = 0.81, 95%CI: 0.67–0.99, *p* = 0.03), respectively.

**Conclusion:**

Our meta-analysis suggested that BDNF rs6265 and rs2049046 polymorphism were associated with common migraine in Caucasian population. Further studies are awaited to update this finding in Asian population and other types of migraine.

## Review

### Background

Migraine, characterized by recurrent headaches accompanying autonomic symptoms, is the 6^th^ leading cause of global years lived with disability (YLDs) according to the Global Burden of Disease Study 2013 [1]. The pathological mechanisms of migraine are very complex. Existing evidences have revealed its association with central sensitization, cortical spreading depression, trigeminovascular system activation and neurogenic inflammation.

Brain-derived neurotrophic factor (BDNF) is the most abundant neurotrophin in the brain [2, 3]. Previous study has recognized it as an important modulator of central and peripheral nociceptive pathways [4, 5]. It distributes in both spinal and supra-spinal levels, contributing to central sensitization [6]. Also it is co-expressed with Calcitonin gene-related peptide (CGRP), an important molecule of migraine, in the trigeminal ganglion neurons [7]. Moreover, significant increase of serum BDNF level was detected in migraine attack patients [8]. Therefore, the alteration in BDNF metabolism may be contribute to the mechanism of migraine.

The Val66Met (rs6265) polymorphism is the most common and studied variant of BDNF gene. It can disrupt the release of mature BNDF and contribute to migraine. Meanwhile, the rs2049046 polymorphism was also found to be associated with migraine. It may influence the transcription of BDNF gene to induce migraine [9, 10].

The association between BDNF gene variant and migraine attracts more and more attention in recent years. However, their roles among the disorder sufferers are still not well established [9–12]. Then we systematically searched and analyzed the available studies to address these issue.

## Methods

### Literature-search strategy

The literature search was performed in October 2016 without restriction of language, region and publication type. PubMed, Web of Science and Cochrane databases were systematically searched to identify all relevant studies. The following terms and their combinations were searched in title and/or abstract: BDNF, brain-derived neurotrophic factor, polymorphism, migraine and headache. The reference lists of included studies and review articles were manually searched to find relevant studies. The search was conducted independently by two of the authors (Cai and Shi). If the same group of participants was studied for more than one times, the latest publication was included.

### Inclusion and exclusion criteria

Studies investigating the association between BNDF polymorphism and migraine were evaluated. The following inclusion criteria were applied to select eligible studies: (1) independent case–control study evaluated the association between BDNF polymorphism and migraine; (2) BDNF rs6265 (G/A) polymorphism and/or rs2049046 (A/T) polymorphism were evaluated; (3) migraine diagnosis should met the International Headache Society (IHS) criteria; (4) Hardy-Weinberg equilibrium (HWE) must be performed; (5) genotype data of cases and controls must be available. Exclusion criteria were as follows: (1) no controls; (2) reviews, comments and animal studies.

### Data extraction and quality assessment

Data from included studies was extracted and summarized independently by two of the authors (Cai and Shi). Any disagreement was resolved by discussion and reexamination. The following information was extracted prospectively: first author, publish year, country, ethnicity, age, sex, number of cases and controls, frequency of available genotype, genotype method and Hardy-Weinberg equilibrium (HWE) evidence in controls.

The quality of included studies was evaluated independently through the Newcastle-Ottawa scale (NOS) by two of the authors (Cai and Shi). NOS is composed of eight assessment items for quality appraisal, with score ranging from 0–9 [13]. According to the NOS scores, the included studies were classified as low-quality study (0–4), moderate-quality study (5–6) and high-quality study (7–9). Any disagreement was resolved by the senior authors (Fang and Zhang).

### Statistical analysis

The strength of the association between BDNF genetic polymorphism and migraine was calculated using odds ratio (OR), with corresponding 95% confidence interval (CI). BDNF rs6265 (G/A) polymorphism and rs2049046 (A/T) polymorphism were evaluated separately in the allele model, dominant model, recessive model and additive model. Heterogeneity among studies was examined through Chi squared-based Q-test and I^2^ test [14, 15]. The heterogeneity difference was regarded significant when *p* < 0.1 in Q test or I^2^ > 50%. If there was heterogeneity among studies, random-effects model (DerSimonian Laird method) was applied to calculate the summary OR, otherwise, the fixed-effects model (Mantel-Haenszel method) was used [16]. The Z-test was used to assess the significance of pooled OR, and *p* < 0.05 was considered significant.

One-way sensitivity analysis was performed to evaluate the influence of a single study on the overall result. Begg’s test and Egger’s test were applied to assess publication bias [17, 18]. All p values were two tailed. All of the meta-analysis were conducted using STATA 12.0 (StataCorp, College Station, TX, USA).

## Results

**Fig. 1:**
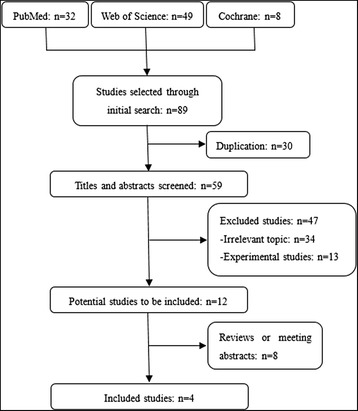
Flow diagram of literature search and study selection

A total of 89 studies were identified through searching in PubMed, Web of Science and Cochrane databases. Four studies with 1598 cases and 1585 controls fulfilling the predefined inclusion criteria were included in the current meta-analysis (Fig. 1). Among them, agreement between two reviewers was 98% for study selection and 95% for quality assessment of studies.

### Characteristics of eligible studies

The characteristics of included studies were shown in Table 1. The genotype and allele frequency of included studies were shown in Table 2. All the included cases were from five Caucasian population groups, two of which came from the Australian-based independent cohorts [10]. The five groups received BDNF rs6265 (G/A) polymorphism analysis, and four of them got rs2049046 (A/T) polymorphism assessment. All SNPs of included studies were in Hardy-Weinberg equilibrium (HWE). The NOS score of the studies were no less than 6, showing good quality. The frequency of rs6265 G allele in BDNF gene was about 80%, which was consistent with previous finding [19].

**Table 1:** Main characteristics of all eligible studies

First author	Year	Country	Ethnicity	Eligible participants (M/F)		Age, mean (SD), years		Genotype method	SNP^a^	HWE	NOS
				Cases	Controls	Cases	Controls				
Salih et al.	2016	Turkey	Caucasian	288 (137/151)	288 (133/155)	31.26 (10.28)	31.53 (8.92)	RT-PCR	1,2	yes	6
Heidi et al.	2014	Australia	Caucasian	277	277	-	-	PCR-RFLP	1,2	yes	7
Heidi et al.	2014	Australian	Caucasian	580	580	-	-	PCR-RFLP	1,2	yes	7
Carolina et al.	2010	Portugal	Caucasian	188 (35/153)	287 (70/217)	36.14 (12.84)	36.42 (12.35)	RT-PCR	1,2	yes	7
Martin et al.	2008	German	Caucasian	265 (43/222)	153 (43/110)	43.6 (13)	64.5 (9.4)	RT-PCR	1	yes	7

*Abbreviation*: *M* male, *F* female, *SNP* single-nucleotide polymorphism, *HWE* Hardy-Weinberg equilibrium, *NOS* Newcastle-Ottawa scale, *PCR*-*RELP* polymerase chain reaction-restricted fragments length polymorphism, *RT*-*PCR* real-time polymerase chain reaction

^a^1 = rs6265; 2 = rs2049046

**Table 2:** Distribution of genotype and allele of BDNF polymorphism between cases and controls

rs number	Source	Group	N	Genotypes (N,freq)			Alleles (N,freq)	
rs6265(G/A)				GG	GA	AA	G	A
	Salih et al.	cases	288	196 (68.1%)	84 (29.2%)	8 (2.8%)	476 (82.6%)	100 (17.4%)
		controls	288	217 (75.3%)	66 (22.9%)	5 (1.7%)	500 (86.8%)	76 (13.2%)
	Heidi et al.	cases	201	131 (65.2%)	64 (31.8%)	6 (3.0%)	326 (81.1%)	76 (18.9%)
		controls	246	171 (69.5%)	68 (27.6%)	7 (2.8%)	410 (83.3%)	82 (16.7%)
	Heidi et al.	cases	411	265 (64.5%)	129 (31.4%)	17 (4.1%)	659 (80.2%)	163 (19.8%)
		controls	546	373 (68.3%)	156 (28.6%)	17 (3.1%)	902 (82.6%)	190 (17.4%)
	Carolina et al.	cases	188	118 (62.8%)	64 (34.0%)	6 (3.2%)	300 (79.8%)	76 (20.2%)
		controls	287	183 (63.8%)	95 (33.1%)	9 (3.1%)	461 (80.3%)	113 (19.7%)
	Martin et al.	cases	265	148 (55.9%)	104 (39.2%)	13 (4.9%)	400 (75.5%)	130 (24.5%)
		controls	153	88 (57.5%)	57 (37.3%)	8 (5.2%)	233 (76.1%)	73 (23.9%)
	Meta-analysis	cases	1353	858 (63.4%)	445 (32.9%)	50 (3.7%)	2161 (79.9%)	545 (20.1%)
		controls	1520	1032 (67.9%)	442 (29.1%)	46 (4.5%)	2506 (82.4%)	534 (17.6%)
rs2049046(A/T)				AA	AT	TT	A	T
	Salih et al.	cases	288	64 (22.2%)	147 (51.0%)	77 (26.7%)	275 (47.7%)	301 (52.3%)
		controls	288	82 (28.5%)	128 (44.4%)	78 (27.1%)	292 (50.7%)	284 (49.3%)
	Heidi et al.	cases	235	46 (19.6%)	115 (48.9%)	74 (31.5%)	207 (44.0%)	263 (56.0%)
		controls	244	60 (24.6%)	128 (52.5%)	56 (23.0%)	248 (50.8%)	240 (49.2%)
	Heidi et al.	cases	549	111 (20.2%)	279 (50.8%)	159 (29.0%)	501 (45.6%)	597 (54.4%)
		controls	561	135 (24.1%)	280 (49.9%)	146 (26.0%)	550 (49.0%)	572 (51.0%)
	Carolina et al.	cases	188	51 (27.1%)	99 (52.7%)	38 (20.2%)	201 (53.5%)	175 (46.5%)
		controls	287	79 (27.5%)	143 (49.8%)	65 (22.6%)	301 (52.4%)	273 (47.6%)
	Meta-analysis	cases	1260	272 (21.6%)	640 (50.8%)	348 (27.6%)	1184 (47.0%)	1336 (53.0%)
		controls	1380	356 (25.8%)	679 (49.2%)	345 (25.0%)	1391 (50.4%)	1369 (49.6%)

*Abbreviation*: *BDNF* brain-derived neurotrophic factor

### Meta-analysis between BNDF gene polymorphism and migraine

#### BDNF rs6265 (G/A) polymorphism in migraine

The main results and heterogeneity between rs6265 (G/A) polymorphism and migraine were shown in Tables 3 and 4. The fixed-effects model was used for all analysis, for their heterogeneity were not significant.

**Table 3:** Heterogeneity among included studies with Chi squared-based Q-test and I^2^ test

rs number	Studies	W Vs M (allele model)		WW + WM Vs MM (dominant model)		WW Vs WM + MM (recessive model)		WW Vs MM (additive model)		WW Vs WM (additive model)	
		p	I^2^	p	I^2^	p	I^2^	p	I^2^	p	I^2^
rs6265(G/A)	5	0.72	0%	0.94	0%	0.78	0%	0.92	0%	0.84	0%
rs2049046(A/T)	4	0.41	0%	0.24	29%	0.72	0%	0.40	0%	0.76	0%

*Abbreviation*: *W* wild allele, *M* mutant allele, *WW* wild homozygote, *WM* heterozygote, *MM* mutant homozygote

**Table 4:** Meta-analysis of the association between BDNF rs6265 and rs2049046 polymorphism and migraine

rs number	Studies	W Vs M (allele model)		WW + WM Vs MM (dominant model)		WW Vs WM + MM (recessive model)		WW Vs MM (additive model)		WW Vs WM (additive model)	
		OR (95% CI)	*p*	OR (95% CI)	*p*	OR (95% CI)	*p*	OR (95% CI)	*p*	OR (95% CI)	*p*
rs6265(G/A)	5	0.86 (0.76, 0.99)	*0.03*	0.85 (0.56, 1.28)	0.42	0.84 (0.72, 0.98)	*0.03*	0.81 (0.53, 1.22)	0.31	0.85 (0.72, 1.00)	*0.04*
rs2049046(A/T)	4	0.88 (0.79, 0.98)	*0.02*	0.89 (0.75, 1.06)	0.18	0.80 (0.67, 0.96)	*0.02*	0.78 (0.62, 0.97)	*0.02*	0.81 (0.67, 0.99)	*0.03*

*Abbreviation*: *W* wild allele, *M* mutant allele, *WW* wild homozygote, *WM* heterozygote, *MM* mutant homozygote

**Fig. 2:**
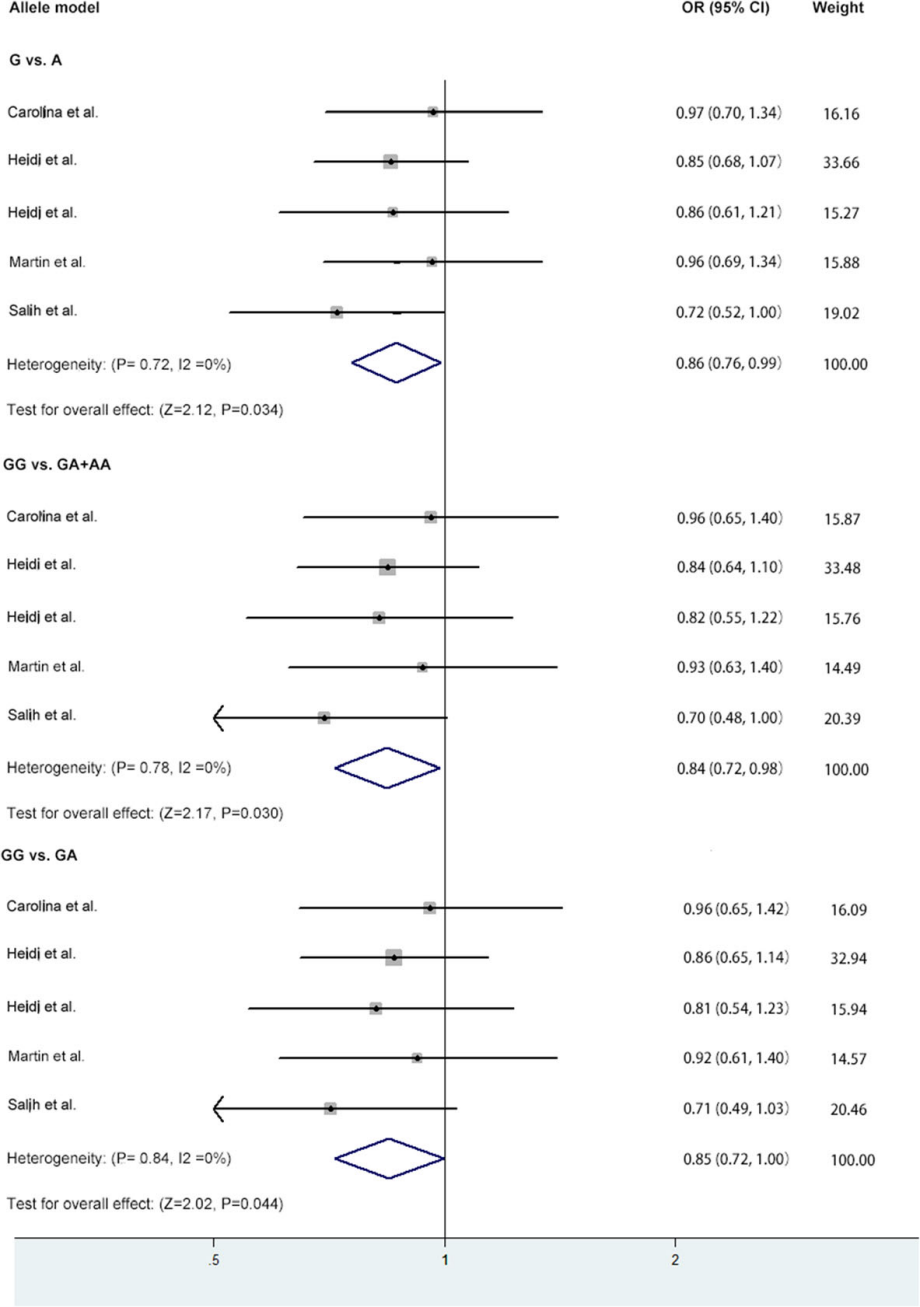
Forest plot of the association between BDNF rs6265 polymorphism and migraine in the allele, recessive and additive model. Abbreviation: BDNF, brain-derived neurotrophic factor

The overall data showed significant association between rs6265 polymorphism and migraine in allele model (OR = 0.86, 95%CI: 0.76–0.99, *p* = 0.03), recessive model (OR = 0.84, 95%CI: 0.72–0.98, *p* = 0.03) and additive model (GG vs GA: OR = 0.85, 95%CI: 0.72–1.00, *p* = 0.04). The association was not significant in dominant model (OR = 0.85, 95%CI: 0.56–1.28, *p* = 0.42) or additive model (GG vs AA: OR = 0.81, 95%CI: 0.53–1.22, *p* = 0.31) (Table 4 and Fig. 2).

#### BDNF rs2049046 (A/T) polymorphism in migraine

The results and heterogeneity between rs2049046 (A/T) polymorphism and migraine were in Tables 3 and 4. Heterogeneity differences were not significant among all the studies. And the fixed-effects model was used.

**Fig. 3:**
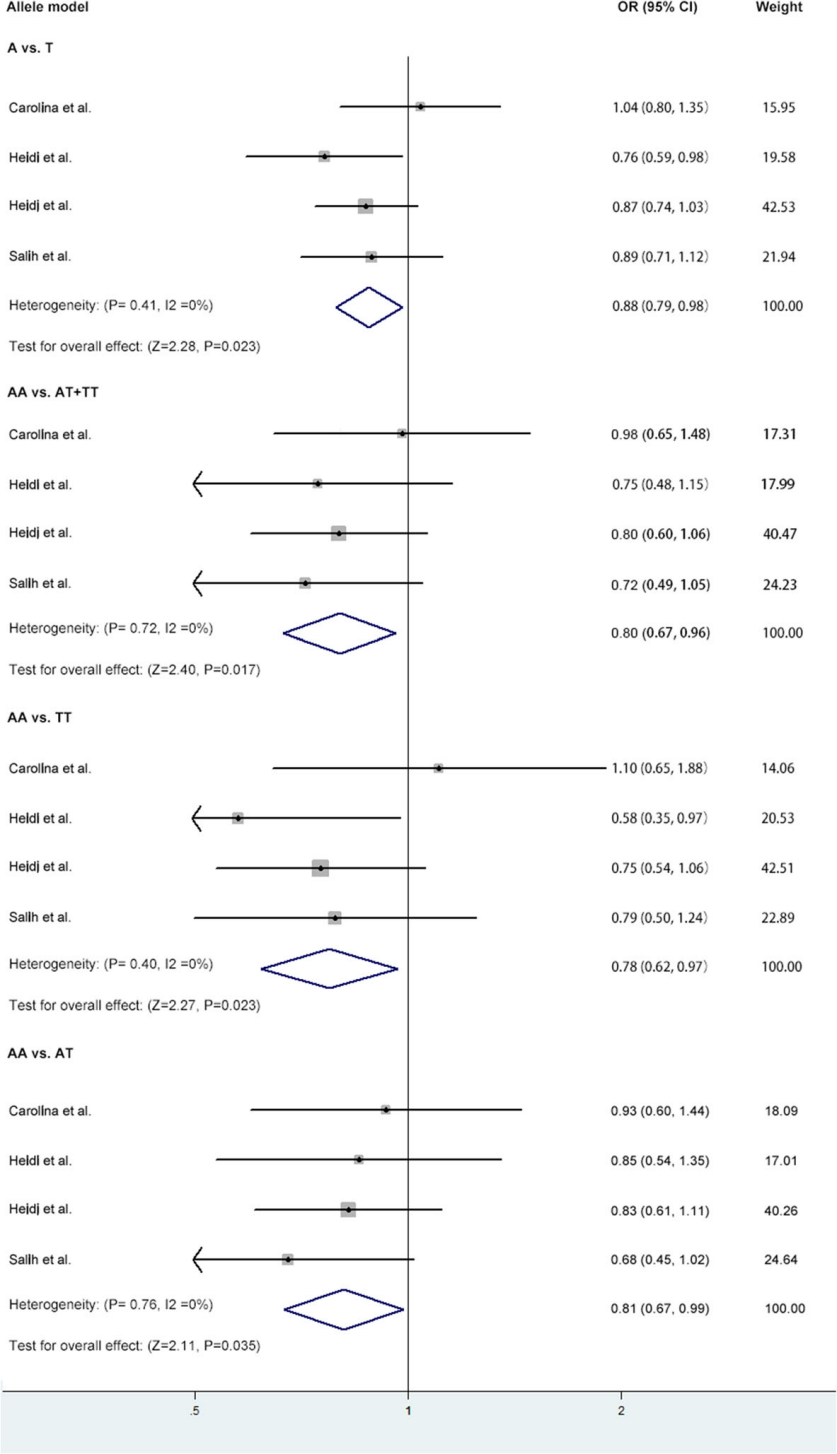
Forest plot of the association between BDNF rs2049046 polymorphism and migraine in the allele, recessive and additive model. Abbreviation: BDNF, brain-derived neurotrophic factor

The data indicated significant association between rs2049046 (A/T) polymorphism and migraine in allele model (OR = 0.88, 95%CI: 0.79–0.98, *p* = 0.02), recessive model (OR = 0.80, 95%CI: 0.67–0.96, *p* = 0.02) and additive model (AA vs TT: OR = 0.72, 95%CI: 0.57–0.92, *p* = 0.008; AA vs AT: OR = 0.81, 95%CI: 0.67–0.99, *p* = 0.03). No significant association was revealed in dominant model (OR = 0.89, 95%CI: 0.75–1.06, *p* = 0.18) (Table 4 and Fig. 3).

### Sensitivity analysis and publication bias

In the sensitivity analysis, there was no change of statistical significance in our analysis when any single study was omitted. Both Begg’s test and Egger’s test indicated that there was no publication bias in our meta-analysis (*P* > 0.05).

## Discussion

This meta-analysis evaluated the association between BDNF gene polymorphism and migraine and showed that BNDF rs6265 (G/A) and rs2049046 (A/T) polymorphism were associated with migraine in Caucasian population.

BDNF, which is associated with the pathogenesis of migraine, shares a wide distribution in central nervous system, such as hippocampus, amygdala, and hypothalamus. It can modulate synaptic plasticity, neurogenesis, neural growth and differentiation [3]. This molecule is synthesized by tyrosine kinase A-positive sensory neurons and acts through TrkB receptors in the nociceptive pathways [20, 21]. Genetic study discovered that BDNF gene is composed of 11 exons that are alternatively spliced to encode different transcripts [22]. Previous studies found that central BDNF can cause neuroplasticity and interact with molecules related to migraine [7, 23]. Serum and cerebrospinal fluid BDNF level elevated in patient with migraine attack. Meanwhile, platelets BDNF level decreased in this sufferers [24, 25]. These findings indicate that alteration of BDNF was responsible for migraine.

The rs6265 polymorphism is a G to A single nucleotide polymorphism (SNP) of BDNF gene at nucleotide 196. It is located in the 5’ pro-protein region of BDNF, resulting in the replacement of the Val66 in the pro-BDNF sequence with a Met. Previous eQTL (expression quantitative trait locus) study has confirmed the association between BDNF Val66Met variant and mRNA level in whole peripheral blood in European [26, 27]. Subjects with Val66 displayed a significantly higher BDNF mRNA expression level compared with subjects with Met66 variant. Also, Met BDNF form cannot be sorted from Golgi to appropriated secretory granules, consequently impairing the secretion function of BDNF [28]. Thus the rs6265 polymorphism can modify the level of BDNF mRNA and the intracellular packaging of pro-BDNF, to finally affect the secretion of mature protein. And the change of BDNF level and neurotrophic-induced neural plasticity could affect the trigeminal pain-related evoked responses and cortical pain processing [29]. Several studies have evaluated the role of rs6265 polymorphism in migraine. However, the intrinsic effects were not thoroughly demonstrated. In this analysis, we confirm this supposition that rs6265 polymorphism may be associated with migraine. The associations between rs6265 polymorphism and this disease in allele, recessive and additive models were all in borderline significant. Consistent with our results, Salih et al.[12] found a borderline significant difference of rs6265 polymorphism in migraine patients, demonstrating a positive correlation between BDNF rs6265 polymorphism and migraine. Di Lorenzo C et al. found that rs6265 polymorphism was associated with monthly drug consumption in medication overuse headache patients, indicating its role in migraine chronicity [30]. Moreover, we found this positive association though enlarging the sample size by combining similar studies. So previous studies with negative results may be due to insufficient sample sizes [9–11].

The rs2049046 polymorphism is located at 5’ end of the BDNF gene, upstream to a region that contains obesity-associated SNPs [31]. It may influence the tissue-specific transcription or levels of BDNF, thus regulating migraine [32]. In the present study, we revealed that individuals carrying rs2049046 T allele might be more susceptive to develop migraine. This finding is consistent with two other studies conducted by Sutherland et al. and Lemos et al. [9, 10]. However, the potential role of rs2049046 polymorphism in the process of BDNF formation and secretion required further investigation. Basic studies on the biological functions of rs2049046 polymorphism and its correlation with migraine are needed.

The studies included were all moderate-high quality, providing a reliable basis for the current analysis. No relative case–control studies were excluded from our analysis. No evidence of public bias was detected in our meta-analysis and the heterogeneity between different studies was insignificant. However, the number of studies included in our meta-analysis was limited. Meanwhile, all the cases were from Caucasian population, and the G allele frequency of BDNF gene rs6265 polymorphism was found to be different between Caucasian (80%) and Asian (56%) population, making our results be unappropriated for Asian countries [19]. Future studies are awaited to update our finding in Asian population. Due to the limited available studies and data, we did not subgroup the patients based on migraine with or without aura. Besides, in our analysis we only evaluated middle-age migraine patients with or with aura, limiting our results to be applied for children with migraine and other types of migraine, such as chronic migraine. Whether the expression of BDNF rs6265 and rs2049046 polymorphism has any difference in different migraine types remains to unclear. In our analysis, we did not obtain raw SNPs data of each study. It limited our further exploration of the combined effect of this two SNPs, which required further study.

So far, several Genome-Wide Association Studies (GWAS) have been perform to evaluate the susceptibility loci of migraine [33–37]. However, the polymorphism of BDNF gene did not reach the significance in them. This difference didn’t indicate the impact of BDNF gene polymorphism was negligible. It may due to the large penalties on significance thresholds in GWAS model (p of association ≤ 10^−7^), leading to the lack of statistical power of BDNF gene. As a candidate-gene association study, our hypothesis arose from the positive association between BDNF and migraine revealed in previous clinical researches. This connection strengthened the reliability of our results. Up to now, with large sample sizes used to analysis, only a small number of susceptible genes has been found to be associated with migraine [38]. Thus candidate-gene association study is still an effective and direct way to illustrate the association between gene and disease.

## Conclusion

In conclusion, our meta-analysis suggested that BDNF rs6265 and rs2049046 polymorphism were associated with common migraine in Caucasian population. And it requires further studies to evaluate the association of BDNF rs6265 and rs2049046 polymorphism with migraine in Asian population and other types of migraine.

## Abbreviations

BDNF: Brain-derived neurotrophic factor
HWE: Hardy-Weinberg equilibrium
SNP: Single nucleotide polymorphism
